# Pathologist-trained machine learning classifiers developed to quantitate celiac disease features differentiate endoscopic biopsies according to modified marsh score and dietary intervention response

**DOI:** 10.1186/s13000-023-01412-x

**Published:** 2023-11-11

**Authors:** Aaron M. Gruver, Haiyan Lu, Xiaoxian Zhao, Angie D. Fulford, Michael D. Soper, Darryl Ballard, Jeffrey C. Hanson, Andrew E. Schade, Eric D. Hsi, Klaus Gottlieb, Kelly M. Credille

**Affiliations:** 1 Clinical Diagnostics Laboratory, Eli Lilly and Company, Lilly Corporate Center, Indianapolis, IN 46285 USA; 2 Wake Forest University School of Medicine, Winston-Salem, NC 27157 USA; 3 Research Informatics, Eli Lilly and Company, Indianapolis, IN 46285 USA; 4 Immunology, Eli Lilly and Company, Indianapolis, IN 46285 USA

**Keywords:** Celiac disease, Marsh classification, Machine learning, Artificial intelligence

## Abstract

**Background:**

Histologic evaluation of the mucosal changes associated with celiac disease is important for establishing an accurate diagnosis and monitoring the impact of investigational therapies. While the Marsh-Oberhuber classification has been used to categorize the histologic findings into discrete stages (i.e., Type 0-3c), significant variability has been documented between observers using this ordinal scoring system. Therefore, we evaluated whether pathologist-trained machine learning classifiers can be developed to objectively quantitate the pathological changes of villus blunting, intraepithelial lymphocytosis, and crypt hyperplasia in small intestine endoscopic biopsies.

**Methods:**

A convolutional neural network (CNN) was trained and combined with a secondary algorithm to quantitate intraepithelial lymphocytes (IEL) with 5 classes on CD3 immunohistochemistry whole slide images (WSI) and used to correlate feature outputs with ground truth modified Marsh scores in a total of 116 small intestine biopsies.

**Results:**

Across all samples, median %CD3 counts (positive cells/enterocytes) from villous epithelium (VE) increased with higher Marsh scores (Type 0%CD3 VE = 13.4; Type 1–3%CD3 VE = 41.9, *p* < 0.0001). Indicators of villus blunting and crypt hyperplasia were also observed (Type 0–2 villous epithelium/lamina propria area ratio = 0.81; Type 3a-3c villous epithelium/lamina propria area ratio = 0.29, *p* < 0.0001), and Type 0–1 crypt/villous epithelial area ratio = 0.59; Type 2–3 crypt/villous epithelial area ratio = 1.64, *p* < 0.0001). Using these individual features, a combined feature machine learning score (MLS) was created to evaluate a set of 28 matched pre- and post-intervention biopsies captured before and after dietary gluten restriction. The disposition of the continuous MLS paired biopsy result aligned with the Marsh score in 96.4% (27/28) of the cohort.

**Conclusions:**

Machine learning classifiers can be developed to objectively quantify histologic features and capture additional data not achievable with manual scoring. Such approaches should be further investigated to improve biopsy evaluation, especially for clinical trials.

**Supplementary Information:**

The online version contains supplementary material available at 10.1186/s13000-023-01412-x.

## Background

Celiac disease is an autoimmune disease that occurs in approximately 1% of individuals worldwide as a result of exposure to gluten proteins [1]. Despite its broad clinical manifestations, as many as 83% of American patients are underdiagnosed and 30–50% fail to respond to a gluten restricted diet [2]. Diagnosis in adults relies on a combination of serology testing and small intestine tissue sampling, and an appropriately obtained duodenal biopsy is critical to establishing an accurate diagnosis prior to life-long diet restriction [1, 3]. The typical histologic manifestations of celiac disease include villus blunting, crypt hyperplasia, and an intraepithelial lymphocytosis [4, 5]. However, the mucosal changes are not evenly distributed throughout the small intestine and are subject to misinterpretation due to improper embedding which highlights the importance of proper tissue biopsy handling [6].

While maintenance of a gluten free diet is the primary treatment approach to celiac disease, the burden of adhering to a restricted diet makes compliance a challenge for many patients. Several pharmacologic interventions are being investigated to provide patients with additional treatment options [7, 8]. The U.S. FDA recently released draft guidance for industry on the development of drugs for adjunctive treatments to help address this unmet need. In this document, the agency suggests sponsors obtain biopsy assessments at screening and following treatment to assess the efficacy and durability of response using a clinically accepted scale [9]. This announcement highlights the importance of being able to reproducibly characterize the histopathologic changes that occur in biopsies collected during investigational trials.

Standardized reporting schemes, such as the Marsh-Oberhuber (i.e. modified Marsh) classification, categorize the histologic findings into distinct types with reference to features observed at specific stages along the disease spectrum [10]. The modified Marsh classification specifically assesses the number of intraepithelial lymphocytes per 100 enterocytes within the most inflamed areas, crypt architecture, and villus length to define 6 categories of disease severity (Type 0-3c). Despite widespread use of this classification and associated scoring approaches, inter-pathologist agreement is suboptimal with mean kappa values between 0.35 and 0.55 [11]. To address the limitations of observer variability, morphometric analyses have been developed to standardize biopsy readouts [6]. Yet while these approaches have made microscopic assessment less subjective, they require significant pathologist effort, often at a centralized laboratory, and are still subject to nuanced sources of observer variability including identification of the villus-crypt border [12].

The ability to overcome inherent limitations with manual scoring, which often relies on categorizing observations using an ordinal scale, is being investigated using artificial intelligence (AI) and machine learning (ML) approaches [13]. ML describes a type of AI that uses computers to algorithmically define patterns in example data and applies that information to new examples for classification or prediction [14]. Technical advances resulting from improved CNNs, graphical processing units, and the commercial availability of user-friendly classification and segmentation tools has led to significant advancements in machine learning applications [14]. The wide application of AI to pathology and laboratory medicine is becoming broadly recognized [14–16], and its value proposition includes reduction of health care costs, improved access, and enhancement of care delivery including reduction of the imprecision that can accompany histological classifications [16]. Recent ML applications have focused on quantitating image features to improve histology-based assessments, identifying the presence of tumor, predicting genetic status, and enhancing disease staging [17–21]. Although prior investigations have assessed computational approaches to the histologic diagnosis of celiac disease using H&E images [22–27], many of these appear to be driven by expertise in the domains of data science, computer programming, and AI engineering. To our knowledge, few have attempted to highlight the ability of pathologists to train, develop, and employ user-friendly ML classifiers to address the practical challenges of developing histology-based solutions despite the increased attention AI tools are receiving in pathology journals [14, 16, 28].

Therefore, in this pilot study we aim to investigate the feasibility of customizing and employing an off-the-shelf, commercially available, histopathology-focused AI software application to quantitatively characterize disease severity using celiac disease as a model system. We test the hypothesis that a pathologist-developed and deployed machine classifier can accurately inform analysis of the associated histopathologic changes. Quantitative microscopic features representing surrogate attributes of the modified Marsh score (e.g., villus blunting, intraepithelial lymphocytosis, and crypt hyperplasia) are evaluated using correlation with the manual assessment as ground-truth. A combined machine learning score produced from individual feature components is derived, and the utility of this histologic classifier is examined using individual paired biopsy sets captured before and after initiation of a gluten restricted diet.

## Methods

### Sample acquisition and slide preparation

Endoscopic biopsy cases from the Wake Forest University pathology laboratory archive were searched for the diagnosis of celiac disease from December 16, 2011 to May 13, 2022, and accompanying pathology reports were reviewed. Eligible, de-identified, archival samples were obtained according to the protocols and procedures approved by the Institutional Review Board of Wake Forest University School of Medicine (IRB #00074626). A total of 116 small intestine biopsies from cases of celiac disease and non-disease controls were obtained. When available, the Marsh score from the original diagnostic evaluation was captured. Twenty-eight paired cases were included (i.e., initial biopsies that were performed to evaluate the presence of celiac disease with a follow-up biopsy to assess response to a gluten-free diet). The formalin-fixed, paraffin embedded (FFPE) blocks were microtomed, producing 5 μm sections for hematoxylin and eosin staining (H&E) and immunohistochemistry (IHC) using a pre-diluted titer of anti-CD3 (2GV6, Roche, Tucson, AZ) or a 1:3000 titer of anti-apolipoprotein A4 (ApoA4; G-8, Santa Cruz, Dallas, TX) on the Ventana DISCOVERY ULTRA platform (Roche) with DISCOVERY ChromoMap DAB detection (Roche). All cases were centrally evaluated using the modified Marsh classification by a board-certified physician specializing in gastrointestinal pathology (HYL) without knowledge of the ML classifier results. Discrepancies with the original diagnostic score were discussed among participating pathologists and a consensus score was produced for study use as needed.

### Slide scanning and machine learning tissue classifier

Specimens were randomized into training or test sets for machine learning classification as described in Table 1. Whole slide images were digitized with the Leica AT2 scanner (Leica Biosystems, Buffalo Grove, IL) at 40x magnification and imported into a HP Z640 with 128GB RAM with an NVIDIA GeForce RTX 2080 Ti graphics processing unit. HALO AI image analysis software (v3.21851.328; Indica labs, Albuquerque, NM) was used to perform training and analysis of specific tissue regions in the duodenal biopsy samples. Prior to image analysis, regions with significant artifact (e.g., tissue folds, air bubbles, excessive mounting media) and tissue areas not suited for analysis were excluded (e.g., improper orientation, inadequate villi). Training cases were selected to represent normal duodenum and a spectrum of modified Marsh scores from 1 to 3c. Ten cases were pathologist annotated to encompass tissue class features (i.e., villous epithelium, crypts, lamina propria, submucosa [including Brunners glands] and white space). A total of 892 annotations, involving a composite area of 21mm^2^, were made on the CD3-labeled WSI. Training of the classifier model was initially performed using the miniNet convolutional neural network (CNN) until convergence to a cross entropy less than 0.15. Tissue classification results were reviewed by two pathologists (KMC, AMG). As performance was largely adequate, but some errors occurred, the set was trained a second time using the DenseNet2 CNN which can learn more complex patterns due to its larger model size. The Densenet2 CNN was trained on the annotations with a resolution of 2 μm/pixel and a minimum object size of 200μm^2^ over 21,000 iterations until a cross entropy of 0.15 was achieved. In cases where the deep learning classifier misidentified regions, corrective annotations were added, and the model was retrained over multiple iterations until the desired performance metrics were achieved. Resultant classification overlay areas on all 116 cases were reviewed and deemed appropriate for further investigation.

**Table 1:** Composition of the study training and test sets

Characteristic	Total Study (*N* = 116)	Training Set (*N* = 10)	Test Set (*N* = 106)
Age category	Count (%)	Count (%)	Count (%)
≤ 21 years	75 (65)	6 (60)	69 (65)
> 21 years	41 (35)	4 (40)	37 (35)
Sex			
Male	58 (50)	6 (60)	52 (49)
Female	58 (50)	4 (40)	54 (51)
Documented biopsy location			
Duodenal bulb	4 (3)	0 (0)	4 (4)
Duodenum, NOS	98 (84)	10 (100)	88 (76)
Small intestine, NOS	14 (12)	0 (0)	14 (13)
Modified Marsh classification			
Type 0^a^	42 (36)	2 (20)	40 (38)
Type 1	10 (9)	1 (10)	9 (8)
Type II	2 (2)	0 (0)	2 (2)
Type IIIa	23 (20)	2 (20)	21 (20)
Type IIIb	37 (32)	5 (50)	32 (30)
Type IIIc	2 (2)	0 (0)	2 (2)

Percentages may not equal 100 due to rounding

^a^Category includes non-disease controls

*NOS* not otherwise specified

### Image analysis routine for CD3 immuno-positive and negative cells

For quantitation of CD3 positive T-cells, an image analysis routine was created in the multiplex IHC HALO module (Multiplex IHC v3.2.3), to identify 3,3′-diaminobenzidine (DAB) cytoplasmic regions of positive lymphocytes and all cell nuclei by hematoxylin with both thresholds set by the pathologist. Nuclear segmentation was performed by the nuclei segmentation AI plug-in classifier. The classified areas of villous epithelium and crypt epithelium identified by the trained DenseNet2 classifier were specifically included as regions for quantitation by the CD3-positive cell image analysis routine. The optimized multiplex IHC algorithm performed on the CD3-labeled images quantified the percent CD3 positive cells within the villous and crypt epithelium separately (CD3 positive cells/enterocytes).

### Analysis of machine learning tissue classifier data and statistics

Area data from the tissue classifier were used to generate the ratio of villous epithelium/lamina propria as a surrogate measure of villus height and the ratio of crypt epithelium/villous epithelium data was used as a surrogate measure of crypt hyperplasia. These proportionate area measurements were quantified and reported in mm^2^. An unpaired, two-tailed t-test was used to compare median values of modified Marsh score categories grouped either according to categories representative of the class type where a relevant histologic change manifests, or as “Type 0–1” and “Type 2–3” categories based upon the cut-off employed in the diagnostic approach for suspected celiac disease in an adult patient on a gluten containing diet [29].

### Generation of machine learning score and analysis method workflow

To produce the combined feature machine learning score (MLS), a linear regression was modelled on the combination of the computed ratio of villous epithelium to lamina propria area (VE ratio), the ratio of crypt epithelium area to villous epithelium area (CE ratio), and the fraction of CD3 positive cells in the villous epithelium (%CD3 VE). The regression was trained based on samples with multiple time points available for a total of 67 data points. The ground-truth modified Marsh scores of 3a/3b/3c were transformed to numeric values of 3.0, 3.333, and 3.666, respectively. The resulting fitted regression model was MLS = 0.872–1.03 (VE ratio) + 0.20 (CE ratio) + 3.92 (%CD3 VE). The overall regression was statistically significant (R^2^ = 0.6156, F(3,63) = 36.2, *p* = 9.86e-14). It was found that the VE ratio and %CD3 VE significantly predicted the Marsh score (*p* = 0.023 and 8.68e-6 respectively), but the CE ratio did not (*p* = 0.1369). This regression model formula was used to evaluate the remaining images of the entire sample set and create continuous MLS outputs from the individual ML features described above. Therefore, the analysis method workflow was to: 1) run the machine learning tissue classifier on the CD3-immunostained WSI to generate tissue region area data, 2) perform the multiplex IHC routine to quantitate CD3-positive and negative cells on the same WSI, 3) collect the CD3 positive cell counts from the villous and crypt epithelium as identified by the tissue classifier, and 4) apply the MLS model to the tissue area and CD3 positive cell quantitative data. For creation of confusion matrices, the continuous machine learning score was converted to an ordinal scale by assigning the closest value number, using values of 3.0/3.333/3.666 for scores of 3a/3b/3c respectively. Data analysis and visualization were performed using Graph Pad Prism (version 9.4.1) or R (version 4.2.2).

## Results

### Classifier assessments of modified marsh score components

**Fig. 1:**
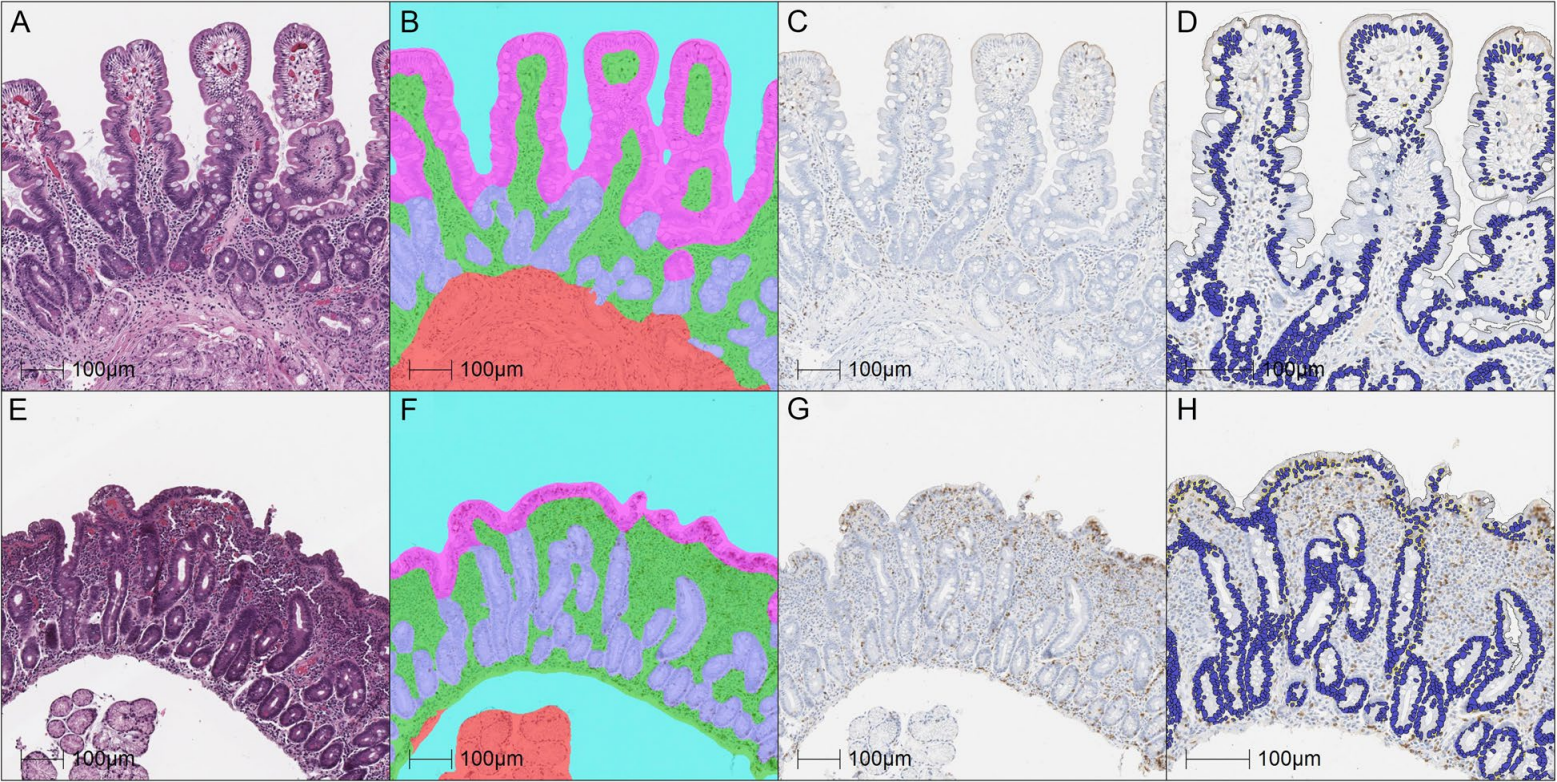
**A** and **E** Normal duodenal tissue and biopsy sample with a modified Marsh score of 3b (H&E), respectively; **B** and **F**) same samples showing the tissue classifier overlay (villous epithelium in pink, crypt epithelium in purple, lamina propria in green and submucosa with Brunners glands in red, white space in blue); **C**) and **G**) CD3 IHC; **D** and **H**) slightly higher magnification showing the CD3 IHC overlay image with nuclei in blue and positive cells with yellow outlines

**Fig. 2:**
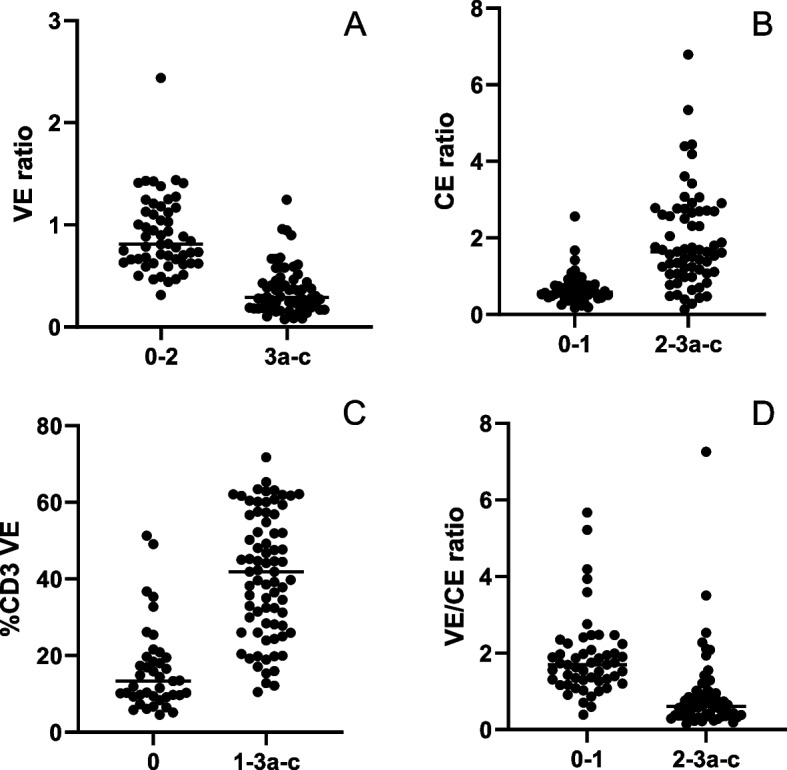
**A** The ratio of the area of villous epithelium (VE) to the area of lamina propria serves as a surrogate for villus height or blunting when comparing by grouped modified Marsh score categories, *p < 0.0001. **B**) The ratio of crypt epithelium (CE) area to villous epithelium area is a surrogate for crypt hyperplasia, *p < 0.0001. **C** The number of CD3 immuno-positive lymphocytes divided by the total number of villous enterocytes was determined, *p < 0.0001. **D** The ratio of the area of villous epithelium to the crypt epithelium is a surrogate for the ratio of villus height to crypt depth, *p < 0.0001

For an initial evaluation of the trained classifiers, we hypothesized that normalized areas of the villous and crypt epithelial compartments could be used as a surrogate for the assessments of villus height and crypt hyperplasia included in the modified Marsh classification. Therefore, quantitative class area measurements associated with the highlighted overlay regions identified from analyzed biopsy tissues (*N* = 116) were exported from the analysis software for comparison with manually obtained scores. Representative classified tissue overlays are shown in comparison with the raw histology images for reference (Fig. 1). Based upon a prior report highlighting the use of IHC to increase the precision of morphometry measurements [12], ApoA4 IHC was performed as an aid to evaluate the ability of the classifier to distinguish the villus-crypt border (Supplemental Fig. 1). Most images demonstrated an overlay consistent with ApoA4 IHC immunoreactivity, though some areas of discordance were observed at the base of villi and a few of the samples failed to stain. As a surrogate for villus height, the total area classified as villous epithelium divided by the total area classified as lamina propria was calculated per sample. Comparison of this ratio to the reference modified Marsh score categories demonstrated a significant correlation of decreased normalized area with an increased manual score indicative of villus blunting (Fig.2A). The median villous epithelium/lamina propria area ratio was 0.81 for Type 0–2 samples and 0.29 for Type 3a-3c, (95% CI − 0.66 to − 0.43, Eta squared = 0.4423, *p* < 0.0001).

A similar approach was used as an assessment of crypt hyperplasia by dividing the total area classified as crypt epithelium by the total area classified as villous epithelium. Comparison of this ratio to the manual score categories demonstrated a significant correlation of increased normalized area with increased Marsh type indicating crypt hyperplasia (Fig. 2B). The median crypt epithelium/villous area ratio was 0.59 for Type 0–1 samples and 1.64 for Type 2–3 samples, (95% CI 0.89 to 1.62, Eta squared = 0.2937, *p* < 0.0001). For the assessment of intraepithelial lymphocytosis, the %CD3 VE (CD3 positive cells/enterocytes) measured specifically in the villous epithelial compartment was employed. Comparison of these values with reference modified Marsh score categories demonstrated a significant correlation of increased %CD3 VE with increased manual score signifying intraepithelial lymphocytosis (Fig. 2C). The median %CD3 VE was 13.4% for Type 0 and 41.9% for Type 1–3, Eta squared = 0.4058, *p* < 0.0001. Once the assessments of villus height and crypt hyperplasia were generated, we investigated the possibility of using these to create a surrogate measure for a villus height to crypt depth ratio. The median values of this relationship were 1.70 for Type 0–1 samples and 0.61 for Type 2-3a-c, 95% CI − 1.38 to − 0.61 Eta squared = 0.1897, *p* < 0.0001 (Fig. 2D). Classifier features plotted by “Type 0–1” and “Type 2–3” categories were also investigated and showed similar results (Supplemental Fig. 2).

The potential for the amount of tissue collected in the endoscopic duodenal biopsy samples to impact classifier performance was assessed by comparing tissue areas for the Type 0–1 and Type 2–3 samples. The median size of the Type 0–1 samples was 5.0 mm^2^ and 7.1 mm^2^ for the Type 2–3 samples, which was not statistically different (Supplemental Fig. 3A). The range of all biopsy sample sizes was 0.6mm^2^ to 28.1mm^2^. Additionally, an assessment of intraepithelial lymphocytosis in younger versus older patients was made. The %CD3 VE was not statistically different in Type 0–1 samples when comparing patients up to 20 years of age (median %CD3 VE = 14.9%) and those 21 years of age and older (median %CD3 VE = 17.4). Similarly, the %CD3 VE was not statistically different in Type 2–3 samples from patients up to 20 years of age compared (%CD3 VE = 46.0%) to those 21 years and older (%CD3 VE = 40.5%) (Supplemental Fig. 3B).

### Creation of a machine learning combined feature score for exploratory celiac disease histology assessments

Because the modified Marsh types can be conceptualized as a unified category based upon assessment of three individual histologic features of celiac disease pathology, we sought to understand whether the individual machine learning outputs could be used to create a useful combined feature MLS. It was apparent from assessment of the individual features that not all had an equally strong correlation with the manually derived modified Marsh score categories. Association was strongest with the villous height measurement and weakest with the assessment of crypt hyperplasia (0.4499 vs. 0.2875, respectively) with the strength of the median %CD3 VE correlation falling in between (0.4012). Therefore, a regression analysis was performed on the subset of tissue samples (*N* = 67) derived from patients who had more than one biopsy submitted for analysis to determine an appropriate weighting scheme for these variables. The resulting MLS can be expressed as either a continuous variable in decimal format or converted to an ordinal scale for direct comparison with the ground-truth modified Marsh scores as described in the Materials and Methods section.

**Fig. 3:**
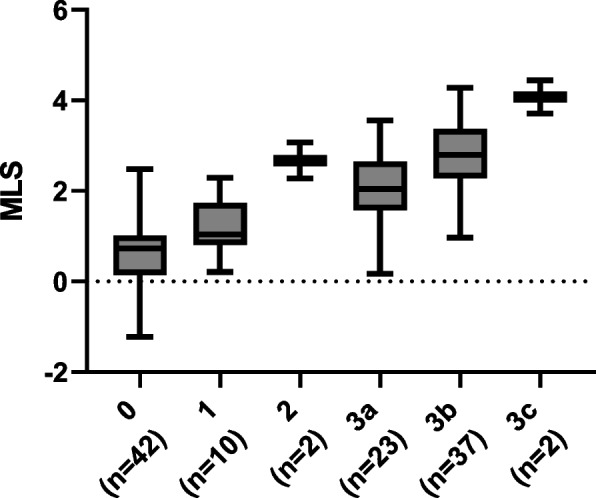
Relationship between the continuous machine learning combined feature score (MLS) and the reference modified Marsh score

Using this combined MLS approach, we next expanded the analysis to examine the relationship between the combined feature score and the reference modified Marsh type across the entire set of 116 samples (Fig. 3). A confusion matrix of these combined feature scores examined by individual Marsh type was also explored (Table 2). A heat map of this confusion matrix demonstrated performance of the converted MLS and ground-truth Marsh score (Supplemental Fig. 4). The median MLS for Type 0 samples was 0.73, while the median values for Type 3a and Type 3b were 2.04 and 2.80, respectively. The median values for Type 2 (2.67) and Type 3c (4.08) aligned with the general observation of increasing MLS with increasing Marsh score; however, the number of samples in these categories are few.

**Table 2:** Confusion matrix of machine learning combined feature score converted to an ordinal scale compared with the modified Marsh type

		Machine Learning Score^a^					
		0	1	2	3a	3b	3c
Modified Marsh Score	**0**	16	20	6	0	0	0
	**1**	2	4	4	0	0	0
	**2**	0	0	1	1	0	0
	**3a**	1	4	9	6	2	1
	**3b**	0	2	13	11	4	7
	**3c**	0	0	0	0	0	2

^a^The machine learning score was converted to an ordinal scale by assigning the closest number, using values of 3.0/3.333/3.666 for scores of 3a/3b/3c respectively

**Fig. 4:**
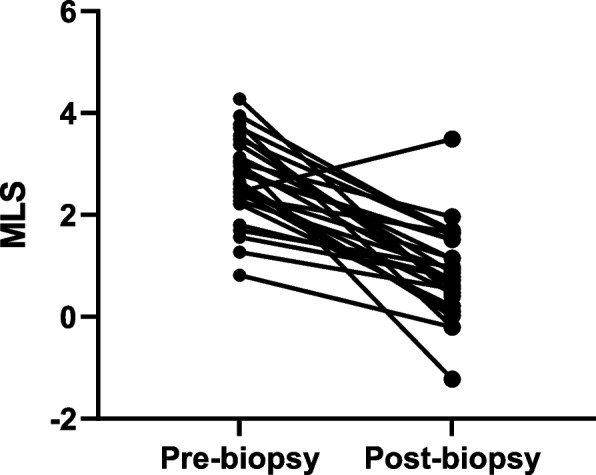
Change in continuous machine learning score (MLS) in 28 patients with matched pairs of duodenal biopsy samples collected from before and after dietary intervention

Because the performance of the combined MLS across both sample groups appeared promising, we sought to investigate the utility of this approach for categorizing the change in histologic features after clinical intervention. For this experiment, the combined feature score was evaluated in the context of paired biopsy samples captured from the same patient both before and after initiation of a gluten restricted diet (Fig. 4). This cohort of 28 patients included 27 matched sample pairs that demonstrated mucosal healing by modified Marsh score (i.e., a manual score response) and one that displayed no change in Marsh score (pair 5). In total, the disposition of the paired biopsy MLS result aligned with the Marsh score in 100% (*N* = 27/27) of the cohort and in 96.4% (N = 27/28) of all matched pairs due to the one patient whose biopsies did not demonstrate a change by Marsh score after diet restriction. (Table 3). Among paired biopsies, the median continuous MLS was 2.72 in “pre-biopsies” and 0.78 in “post-biopsies” with a range of 0.82–4.28 and − 1.23-3.49 in ML scores, respectively.

**Table 3:** Evaluation of paired-biopsy samples

Matched Pair	Modified Marsh Score			Continuous Machine Learning Score		
	Pre- Biopsy	Post- Biopsy	Manual Score Response	Pre- Biopsy	Post- Biopsy	Alignment with Manual Score
1	3b	0	Yes	3.95	1.63	Yes
2	3a	0	Yes	0.82	−0.20	Yes
3	3b	0	Yes	2.44	0.11 4	Yes
4	3b	1	Yes	2.22	0.21	Yes
5	3a	3a	No	2.46	3.49	No
6	3b	0	Yes	3.78	−0.21	Yes
7	3b	0	Yes	2.85	1.52	Yes
8	3a	0	Yes	2.65	0.02	Yes
9	3b	0	Yes	2.44	1.15	Yes
10	1	0	Yes	1.57	0.78	Yes
11	3b	3a	Yes	3.48	1.65	Yes
12	3b	0	Yes	3.56	0.61	Yes
13	3b	1	Yes	2.80	1.51	Yes
14	3a	0	Yes	3.56	0.56	Yes
15	3a	0	Yes	1.27	0.55	Yes
16	2	1	Yes	2.28	0.93	Yes
17	3b	0	Yes	2.36	1.65	Yes
18	3b	0	Yes	4.28	0.40	Yes
19	3a	0	Yes	2.60	0.00	Yes
20	3b	0	Yes	1.70	0.98	Yes
21	3c	3b	Yes	3.71	1.70	Yes
22	3a	0	Yes	1.80	0.78	Yes
23	3b	0	Yes	3.06	−1.23	Yes
24	3b	0	Yes	2.96	0.71	Yes
25	3a	0	Yes	2.51	0.47	Yes
26	3b	0	Yes	3.38	0.87	Yes
27	3b	3a	Yes	3.02	1.96	Yes
28	3b	0	Yes	3.13	1.13	Yes

### Post-analysis assessment of discordant cases among paired biopsy samples

While most of the machine learning combined feature scores correlated with their associated manually derived modified Marsh results, some discrepancies were noted upon post-analysis assessment. As described in the regression analysis, the directional changes in the %CD3 VE data strongly aligned with ground truth Marsh scores in the paired samples that responded to diet restriction with only one case failing to show a similar directional response in %CD3 VE to the improvement in Marsh score. In that case (pair 28), the other features of surrogate villus height and crypt size aligned appropriately to the Marsh scores but the weight of the %CD3 VE affected the MLS. Similarly, only one case did not show a directional alignment in treatment response in the villus height surrogate feature. The weakest surrogate feature, crypt area, was discordant in 27% of the responding pairs. The classifier overlays for those cases were reviewed again and in most, no cause for the non-alignment was observed, such as errors in the classifier assignments. In some cases, it appeared that one possible cause could be related to the small size of the sample and/or sub-optimal orientation of the pre-biopsy resulting in minimal sampling of the crypts (pair 17).

## Discussion

Histologic assessment of small intestine biopsies is a critical tool for the diagnosis of celiac disease and has been used as a co-primary endpoint in the assessment of investigational therapies [30]. Yet, significant observer variability exists among pathologists when using traditional scoring schemes [11]. The current availability of user-friendly, histopathology-focused AI software presents a unique opportunity for pathologists to become more engaged with advancements in computer vision that are rapidly impacting the practice of medicine. In this study, we developed pathologist-trained machine learning classifiers with commercially available, user-friendly, AI software to characterize the histopathologic features of celiac disease from standard immunohistochemical-stained tissue sections. While use of the miniNet network accurately classified a majority of features appropriately, ultimately the denseNet2 classifier was used for this pilot study given its superior performance as inferred from pathologist review of the resulting overlays. The feasibility of this approach was tested by characterizing a set of paired biopsies before and after dietary intervention to mimic a retrospective exploratory assessment conducted in the setting of a clinical trial. Comparison of this machine learning method to the modified Marsh scores was favorable, with correlation observed across Marsh types, and > 90% concordance with manual score outcomes among matched biopsies in this limited sample set.

Machine learning is becoming an important tool to augment the traditional microscopic assessment of histology images across multiple disciplines [21, 31, 32]. Primary advantages of ML-assisted pathologist evaluations include: 1) amelioration of the intra−/inter-observer variability associated with manual scoring approaches, and 2) generation of objective, quantitative results with which subtle changes in the histologic features of biopsy samples may be measured. While Level I evidence generated from a prospective clinical trial is not yet publicly available, these themes intuitively span multiple tissue types and classification systems. For example, a pixel level quantitative assessment of fibrosis to assess disease staging for non-alcoholic steatohepatitis (NASH) is being investigated as an alternative to the ordinal scoring system developed by the NASH Clinical Research Network [21]. The benefit of this approach was retrospectively demonstrated to reduce placebo effect and increase treatment response compared to the manual scores produced by a central pathologist. We hypothesize a similar finding could be observed from the use of ML classifiers for the assessment of celiac disease given the degree to which quantitative machine learning scoring systems would potentially facilitate detection of more subtle histologic changes in the context of adjunctive treatment investigations. The expansive nature of data acquired from cell and tissue based models, for example those that focus on reporting human interpretable features comprising > 600 computer vision derived outputs [18, 32], illustrates the possibility for machine learning to transform the way pathologists develop histology based scoring systems and invites discovery of relationships not apparent with traditional scoring methodology.

The first reports of the use of AI in the evaluation of celiac disease histopathology were published in 2019 through 2022. An initial study utilizing WSI of celiac disease biopsies, unaffected duodenal tissue, and non-specific duodenitis applied a weakly supervised approach by assigning pathologist-derived diagnoses at the level of the image on a set of example cases that then were used to train the CNN to predict the diagnoses of future test cases, resulting in a high accuracy within the test set [23]. At that same time, several groups used similar approaches to compare and predict diagnoses of celiac disease versus normal duodenum [33], celiac disease by modified Marsh score [24], celiac disease versus environmental enteropathy [34–36], and celiac disease by modified Marsh score versus environmental enteropathy [21] using CNNs, resulting in high accuracy in their limited study sets.

Several of these prior publications also employed techniques to address explainability of the algorithm or the “black box” problem by using activation mapping and other methods to apply heat maps to the images that highlight those parts of the tissues most important in predicting the diagnosis. The promise of this approach is that pathologist review of these highlighted images can provide new information on the biology and pathology of these diseases. The Syed publication in 2021 also began to employ ensemble methods, combining algorithms created by different CNNs, to improve accuracy. One group during this period developed algorithms to discriminate modified Marsh scores in celiac disease histology using conventional machine learning classifiers, such as support vector machines and Adaboost rather than CNNs, with similar accuracy in their results [25]. A recent report focused on diagnosing celiac disease split by modified Marsh scores versus environmental enteropathy using a CNN derived algorithm, and also incorporating manual morphometry techniques to obtain cell counts in villi, crypts and lamina propria and villi height- crypt depth measurements [37]. The application of the CNNs in the Halo AI software in our study uniquely focused not on training algorithms to predict diagnoses, but to generate tools to more easily create surrogates for onerously derived manual morphometric data, difficult to measure features such as villus height to crypt depth, and more detailed continuous data to describe celiac disease features beyond ordinal scores.

This pilot study is not without limitations which include a relatively small sample size collected from a single institution, and the inability to test biopsy samples collected as part of a clinical trial to directly measure adjunctive treatment effect. Class imbalance in the dataset (i.e., relatively few cases of Marsh Type 2 and Type 3c) may have influenced the linear regression by producing a reduced weight for the CE ratio. While this could have resulted in a tighter range of MLS values than might be observed in a larger sample set, it did not appear to diminish the ability of the model to resolve subcategory changes in paired biopsies before and after dietary gluten restriction (e.g., matched pairs 11, 21, and 27). One other concern was that by determining the %CD3 positive cells across the entire villous epithelium, we could possibly “dilute” or overlook focal areas of high intraepithelial lymphocytosis and those cases would receive lower %CD3 VE as compared to manual scores. Overall, most Type 2-3c samples demonstrated multifocal to diffuse intraepithelial lymphocytosis so that the %CD3 VE was representative of the samples. However, an “undercount” was noted in one sample (pair 28 pre-biopsy), affecting the MLS and resulting in non-alignment with the Marsh score. This underscores the fact that these ML quantitative methods are adjuncts to the pathologist review and are not replacements. Similarly, these ML classifiers are applied to samples regardless of their appropriate orientation. In this case, each WSI was reviewed by a pathologist and only the acceptable samples were selected for analysis. In the future, more sophisticated ML tools may be developed that will also perform more complex assessments such as small intestine endoscopic biopsy orientation.

Strengths of the study include the use of samples comprising a spectrum of celiac disease severity and the use of immunohistochemical stains to evaluate individual cellular (e.g., CD3) and tissue specific (e.g., ApoA4) features of the resulting CNN overlay images to confirm classifier accuracy. The observation that the highest correlations between individual ML components and modified Marsh score were the VE ratio and IEL measured by %CD3 is not surprising. The ability of CD3 IHC to assist in the identification of intraepithelial lymphocytosis has long been recommended [10], and the use of 40 lymphocytes as the cut-off value for IEL in the modified Marsh classification is the only numerically defined feature. Prior studies have also suggested that lymphocytic analysis is less impacted by plane of sectioning than villus or crypt architecture, both of which are impacted by biopsy orientation [6]. Unlike the villus assessment, which is represented in 3 categories of “mild atrophy”, “moderate atrophy”, and “marked atrophy” with increasing disease type, the crypt evaluation is characterized as either “normal” or “hyperplastic/hypertrophic” in the modified Marsh scheme. It was interesting to observe that the machine learning surrogate of crypt hyperplasia from the present study was also the feature with the weakest correlation to the ground truth Marsh scores, indicating that classifier performance for this feature aligns with the real-world experience of crypt hyperplasia being a relatively poor indicator of celiac disease.

## Conclusions

In summary, we describe a novel approach for using pathologist-trained machine learning classifiers for the assessment of celiac disease biopsies. While other viable approaches are examining the ability to classify disease features from H&E stained tissues [27], this method demonstrated the feasibility of customizing off-the-shelf, AI software to objectively quantify histopathologic features from routinely processed and immune-stained sections available in the standard clinical laboratory. Future work with larger cohorts will be needed to understand the impact of disease heterogeneity on classifier performance and explore model generalizability. Machine learning models for the characterization of celiac disease should be further investigated to improve biopsy evaluation, assess disease severity, and characterize response to therapeutic interventions.

### Supplementary Information

**Additional file 1: Supplemental Fig. 1.** A) APOA4 IHC to help define the villous epithelium was applied to normal duodenal tissue and shows cytoplasmic labeling of villous epithelial cells that is often strongest at the villi tips, lessening down the villus and becomes weak and sometimes discontinuous at the opening of the crypts (arrows). B) CD3 IHC of the same sample and its’ tissue classifier overlay with the pink color marking villous epithelium and the purple marking the crypt epithelium. The classifier generally aligns with the APOA4 IHC, with some deeper extension into the crypts. C) APOA4 IHC applied to a celiac disease modified Marsh score 3b sample to define the flattened villous epithelium and D) the matching CD3 section with its classifier overlay.

**Additional file 2: Supplemental Fig. 2.** A) The ratio of the area of villous epithelium (VE) divided by the area of lamina propria serves as a surrogate for villus height or blunting when comparing by grouped modified Marsh score categories, *p < 0.0001. B) The ratio of crypt epithelium (CE) area to the villous epithelium area is a surrogate for crypt hyperplasia, *p < 0.0001. C) The number of CD3 immuno-positive lymphocytes divided by the total number of villous enterocytes was determined, *p < 0.0001. D) The ratio of the area of villous epithelium divided by the crypt epithelium is a surrogate for the ratio of villus height to crypt depth, *p < 0.0001

**Additional file 3: Supplemental Fig. 3.** A) The area of the duodenal tissue biopsy samples was not statistically different across grouped modified Marsh scores. The median area for Type 0–1 scores was 5.0 mm^2^ and for Type 2-3a-c scores was 7.1 mm^2^. B) The number of CD3 immuno-positive lymphocytes divided by the total number of villous enterocytes was not statistically different by grouped modified Marsh scores and patient age stratified above and below 21 years.

**Additional file 4: Supplemental Fig. 4.** Confusion matrix heat map showing performance of converted machine learning scores and modified Marsh type.

## Data Availability

The dataset used during the current study are available from the corresponding author upon reasonable request.

## Abbreviations

CNN: Convoluted neural network
WSI: Whole slide images
IEL: Intra-epithelial lymphocytes
VE: Villous epithelium
CE: Crypt epithelium
MLS: Machine learning score
AI: Artificial intelligence
ML: Machine learning.

## References

[1] Lebwohl B, Sanders DS, Green PHR (2018). Coeliac disease. Lancet.

[2] Anonymous. Celiac Disease: Fast Facts. www.beyondceliac.org Accessed 15 May 2023.

[3] Singh P, Arora A, Strand TA, Leffler DA, Catassi C, Green PH, Kelly CP, Ahuja V, Makharia GK (2018). Global prevalence of celiac disease: systematic review and Meta-analysis. Clin Gastroenterol Hepatol.

[4] Thurlbeck WM, Benson JA, Dudley HR (1960). The histopathologic changes of sprue and their significance. Am J Clin Pathol.

[5] Serra S, Jani PA (2006). An approach to duodenal biopsies. J Clin Pathol.

[6] Taavela J, Koskinen O, Huhtala H, Lahdeaho ML, Popp A, Laurila K, Collin P, Kaukinen K, Kurppa K, Maki M (2013). Validation of morphometric analyses of small-intestinal biopsy readouts in celiac disease. PLoS One.

[7] Alhassan E, Yadav A, Kelly CP, Mukherjee R (2019). Novel nondietary therapies for celiac disease. Cell Mol Gastroenterol Hepatol.

[8] Gottlieb K, Dawson J, Hussain F, Murray JA (2015). Development of drugs for celiac disease: review of endpoints for phase 2 and 3 trials. Gastroenterol Rep.

[9] U.S. FDA. Celiac Disease: Developing Drugs for Adjunctive Treatment to a Gluten-Free Diet Guidance for Industry *DRAFT GUIDANCE*. FDA-2021-D-1238. 2022. Internet, Accessed 8 Jun 2023. Available from https://www.fda.gov/regulatory-information/search-fda-guidance-documents/celiac-disease-developing-drugs-adjunctive-treatment-gluten-free-diet.

[10] Oberhuber G, Granditsch G, Vogelsang H (1999). The histopathology of coeliac disease: time for a standardized report scheme for pathologists. Eur J Gastroenterol Hepatol.

[11] Corazza GR, Villanacci V, Zambelli C, Milione M, Luinetti O, Vindigni C, Chioda C, Albarello L, Bartolini D, Donato F (2007). Comparison of the interobserver reproducibility with different histologic criteria used in celiac disease. Clin Gastroenterol Hepatol.

[12] Taavela J, Viiri K, Valimaki A, Sarin J, Salonoja K, Maki M, Isola J (2021). Apolipoprotein A4 defines the villus-crypt border in duodenal specimens for celiac disease morphometry. Front Immunol.

[13] Niazi MKK, Parwani AV, Gurcan MN (2019). Digital pathology and artificial intelligence. Lancet Oncol.

[14] Harrison JH, Gilbertson JR, Hanna MG, Olson NH, Seheult JN, Sorace JM, Stram MN (2021). Introduction to artificial intelligence and machine learning for pathology. Arch Pathol Lab Med.

[15] Rakha EA, Toss M, Shiino S, Gamble P, Jaroensri R, Mermel CH, Chen PC (2021). Current and future applications of artificial intelligence in pathology: a clinical perspective. J Clin Pathol.

[16] Paranjape K, Schinkel M, Hammer RD, Schouten B, Nannan Panday RS, Elbers PWG, Kramer MHH, Nanayakkara P (2021). The value of artificial intelligence in laboratory medicine. Am J Clin Pathol.

[17] Campanella G, Hanna MG, Geneslaw L, Miraflor A, Werneck Krauss Silva V, Busam KJ, Brogi E, Reuter VE, Klimstra DS, Fuchs TJ (2019). Clinical-grade computational pathology using weakly supervised deep learning on whole slide images. Nat Med.

[18] Diao JA, Wang JK, Chui WF, Mountain V, Gullapally SC, Srinivasan R, Mitchell RN, Glass B, Hoffman S, Rao SK, Maheshwari C, Lahiri A, Prakash A, McLoughlin R, Kerner JK, Resnick MB, Montalto MC, Khosla A, Wapinski IN, Beck AH, Elliott HL, Taylor-Weiner A (2021). Human-interpretable image features derived from densely mapped cancer pathology slides predict diverse molecular phenotypes. Nat Commun.

[19] Fu Y, Jung AW, Torne RV, Gonzalez S, Vohringer H, Shmatko A, Yates LR, Jimenez-Linan M, Moore L, Gerstung M (2020). Pan-cancer computational histopathology reveals mutations, tumor composition and prognosis. Nat Can.

[20] Kather JN, Heij LR, Grabsch HI, Loeffler C, Echle A, Muti HS, Krause J, Niehues JM, Sommer KAJ, Bankhead P, Kooreman LFS, Schulte JJ, Cipriani NA, Buelow RD, Boor P, Ortiz-Bruchle NN, Hanby AM, Speirs V, Kochanny S, Patnaik A, Srisuwananukorn A, Brenner H, Hoffmeister M, van den Brandt PA, Jager D, Trautwein C, Pearson AT, Luedde T (2020). Pan-cancer image-based detection of clinically actionable genetic alterations. Nat Can.

[21] Taylor-Weiner A, Pokkalla H, Han L, Jia C, Huss R, Chung C, Elliott H, Glass B, Pethia K, Carrasco-Zevallos O, Shukla C, Khettry U, Najarian R, Taliano R, Subramanian GM, Myers RP, Wapinski I, Khosla A, Resnick M, Montalto MC, Anstee QM, Wong VW, Trauner M, Lawitz EJ, Harrison SA, Okanoue T, Romero-Gomez M, Goodman Z, Loomba R, Beck AH, Younossi ZM (2021). A machine learning approach enables quantitative measurement of liver histology and disease monitoring in NASH. Hepatology.

[22] Kowsari K, Sali R, Ehsan L, Adorno W, Ali A, Moore S, et al. HMIC: Hierarchical medical image classification, a deep learning approach. Information. 2020:11(6):318. 10.3390/info11060318 PMC8346231 34367687

[23] Syed S, Al-Boni M, Khan MN, Sadiq K, Iqbal NT, Moskaluk CA, Kelly P, Amadi B, Ali SA, Moore SR, Brown DE (2019). Assessment of machine learning detection of environmental enteropathy and celiac disease in children. JAMA Netw Open.

[24] Wei JW, Wei JW, Jackson CR, Ren B, Suriawinata AA, Hassanpour S (2019). Automated detection of celiac disease on duodenal biopsy slides: a deep learning approach. J Pathol Inform.

[25] Sali R, Ehsan L, Kowsari K, Khan M, Moskaluk CA, Syed S, Brown DE (2019). CeliacNet: celiac disease severity diagnosis on duodenal histopathological images using deep residual networks. Proceedings (IEEE Int Conf Bioinformatics Biomed).

[26] Koh JEW, De Michele S, Sudarshan VK, Jahmunah V, Ciaccio EJ, Ooi CP, Gururajan R, Gururajan R, Oh SL, Lewis SK, Green PH, Bhagat G, Acharya UR (2021). Automated interpretation of biopsy images for the detection of celiac disease using a machine learning approach. Comput Methods Prog Biomed.

[27] Griffin M, Gruver AM, Shah C, Wani Q, Fahy D, Khosla A, Krirkup C, Borders D, Brosnan-Cashman J, Fulford A, Credille KM, Najdawi CF, Gottlieb K. Fully automated histological classification of cell types and tissue regions of celiac disease is feasible and correlates with the Marsh Score [Tu1352). Poster presented at DDW2023, Chicago, IL, 2023.

[28] Patey-Mariaud De Serre N, Cellier C, Jabri B, Delabesse E, Verkarre V, Roche B, Lavergne A, Briere J, Mauvieux L, Leborgne M, Barbier JP, Modigliani R, Matuchansky C, MacIntyre E, Cerf-Bensussan N, Brousse N (2000). Distinction between coeliac disease and refractory sprue: a simple immunohistochemical method. Histopathology.

[29] Ciarán P, Kelly M. Diagnostic approach for suspected celiac disease in an adult patient on a gluten containing diet. www.uptodate.com. Accessed 10 June 2023.

[30] Schuppan D, Maki M, Lundin KEA, Isola J, Friesing-Sosnik T, Taavela J, Popp A, Koskenpato J, Langhorst J, Hovde O, Lahdeaho ML, Fusco S, Schumann M, Torok HP, Kupcinskas J, Zopf Y, Lohse AW, Scheinin M, Kull K, Biedermann L, Byrnes V, Stallmach A, Jahnsen J, Zeitz J, Mohrbacher R, Greinwald R, Group CECT (2021). A randomized trial of a transglutaminase 2 inhibitor for celiac disease. N Engl J Med.

[31] Raciti P, Sue J, Retamero JA, Ceballos R, Godrich R, Kunz JD, et al. Clinical validation of artificial intelligence-augmented pathology diagnosis demonstrates significant gains in diagnostic accuracy in prostate cancer detection. Arch Pathol Lab Med. 2023;147(10):1178-85. 10.5858/arpa.2022-0066-OA 36538386

[32] Najdawi F, Sucipto K, Mistry P, Hennek S, Jayson CKB, Lin M, Fahy D, Kinsey S, Wapinski I, Beck AH, Resnick MB, Khosla A, Drage MG (2023). Artificial intelligence enables quantitative assessment of ulcerative colitis histology. Mod Pathol.

[33] Denholm J, Schreiber BA, Evans SC, Crook OM, Sharma A, Watson JL, Bancroft H, Langman G, Gilbey JD, Schonlieb CB, Arends MJ, Soilleux EJ (2022). Multiple-instance-learning-based detection of coeliac disease in histological whole-slide images. J Pathol Inform.

[34] Kowsari K, Sali R, Khan MN, Adorno W, Ali SA, Moore SR, Amadi BC, Kelly P, Syed S, Brown DE (2019). Diagnosis of celiac disease and environmental enteropathy on biopsy images using color balancing on convolutional neural networks. Proc Futur Technol Conf FTC.

[35] Al Boni M, Syed S, Ali A, Moore SR, Brown DE (2019). Duodenal biopsies classification and understanding using convolutional neural networks. AMIA Jt Summits Transl Sci Proc.

[36] Syed S, Ehsan L, Shrivastava A, Sengupta S, Khan M, Kowsari K, Guleria S, Sali R, Kant K, Kang SJ, Sadiq K, Iqbal NT, Cheng L, Moskaluk CA, Kelly P, Amadi BC, Asad Ali S, Moore SR, Brown DE (2021). Artificial intelligence-based analytics for diagnosis of small bowel enteropathies and black box feature detection. J Pediatr Gastroenterol Nutr.

[37] Khan M, Jamil Z, Ehsan L, Zulqarnain F, Srivastava S, Siddiqui S, Fernandes P, Raghib M, Sengupta S, Mujahid Z, Ahmed Z, Idrees R, Ahmed S, Umrani F, Iqbal N, Moskaluk C, Raghavan S, Cheng L, Moore S, Ali SA, Iqbal J, Syed S (2023). Quantitative morphometry and machine learning model to explore duodenal and rectal mucosal tissue of children with environmental enteric dysfunction. Am J Trop Med Hyg.

