# Supplementary figures and images for: Pathologist-trained machine learning classifiers developed to quantitate celiac disease features differentiate endoscopic biopsies according to modified marsh score and dietary intervention response
Source: Diagn Pathol. 2023 Nov 11;18:122. doi: 10.1186/s13000-023-01412-x (PMC10638821; doi:10.1186/s13000-023-01412-x)

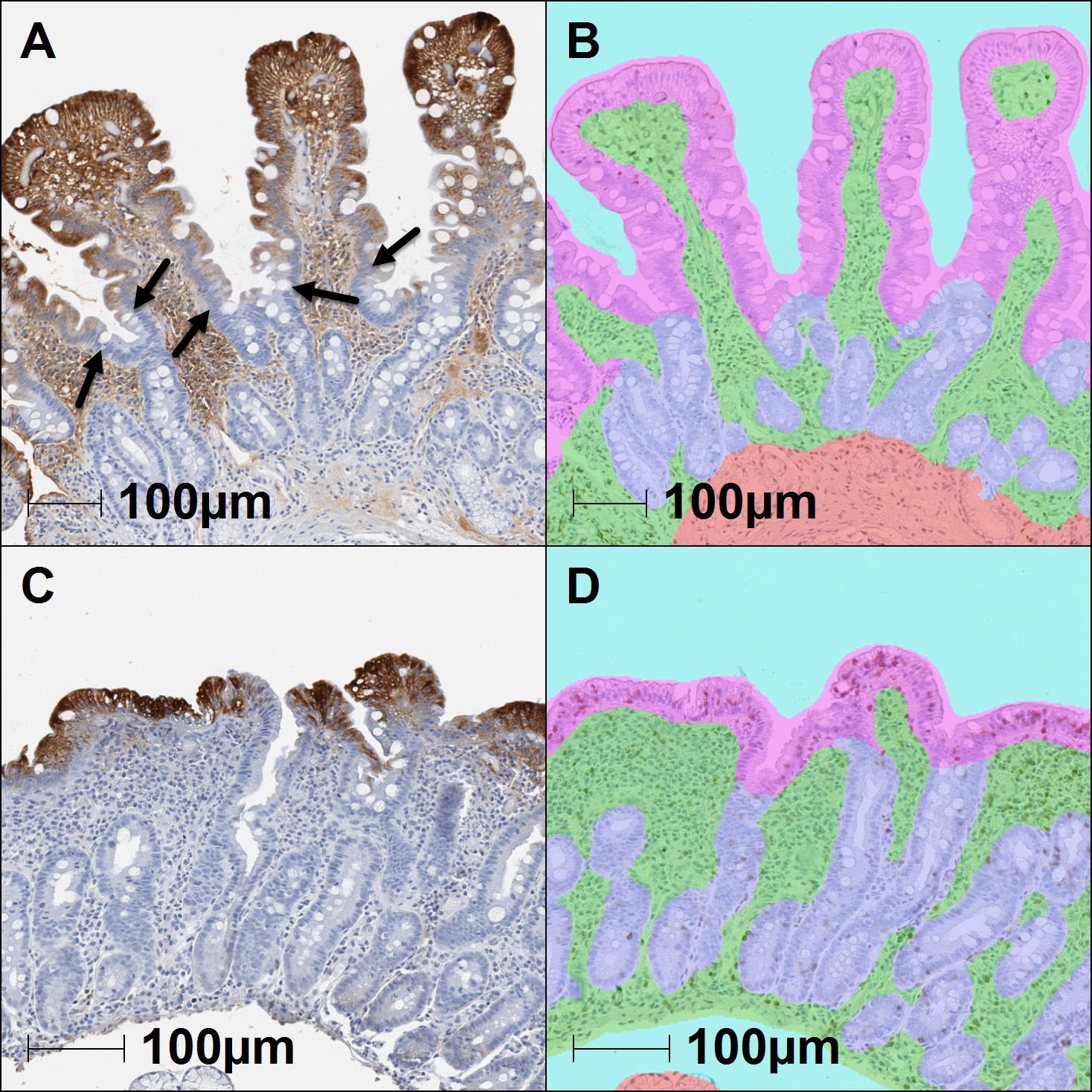

Supplement: Supplementary file 1 — Additional file 1: Supplemental Fig. 1. A) APOA4 IHC to help define the villous epithelium was applied to normal duodenal tissue and shows cytoplasmic labeling of villous epithelial cells that is often strongest at the villi tips, lessening down the villus and becomes weak and sometimes discontinuous at the opening of the crypts (arrows). B) CD3 IHC of the same sample and its’ tissue classifier overlay with the pink color marking villous epithelium and the purple marking the crypt epithelium. The classifier generally aligns with the APOA4 IHC, with some deeper extension into the crypts. C) APOA4 IHC applied to a celiac disease modified Marsh score 3b sample to define the flattened villous epithelium and D) the matching CD3 section with its classifier overlay. [file 13000_2023_1412_MOESM1_ESM.jpg]

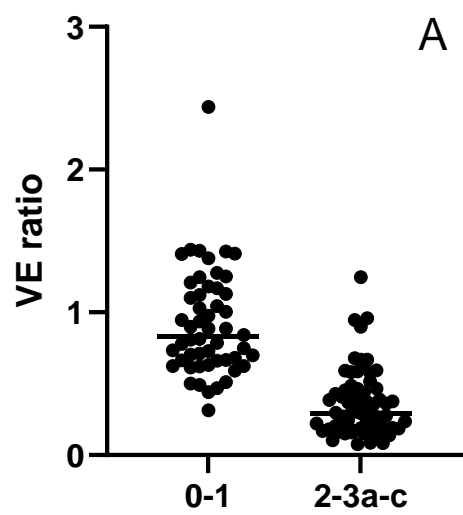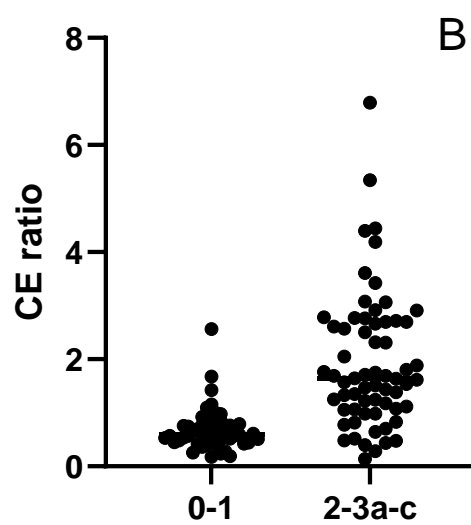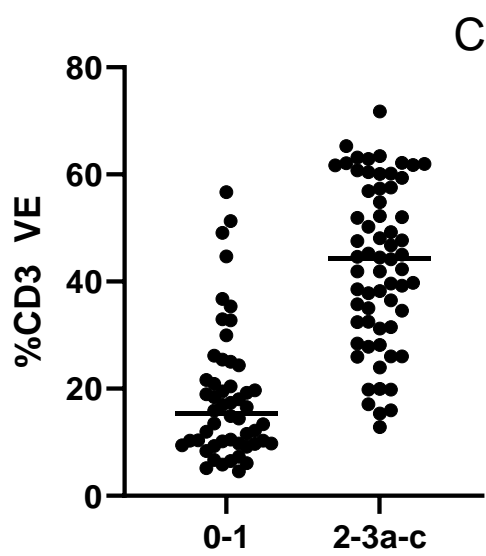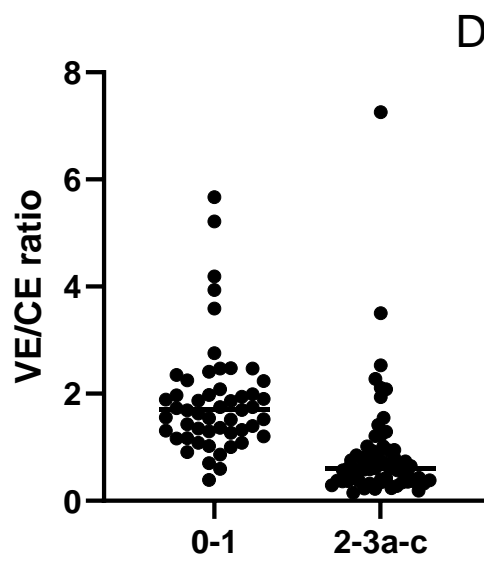

Supplement: Supplementary file 2 — Additional file 2: Supplemental Fig. 2. A) The ratio of the area of villous epithelium (VE) divided by the area of lamina propria serves as a surrogate for villus height or blunting when comparing by grouped modified Marsh score categories, *p < 0.0001. B) The ratio of crypt epithelium (CE) area to the villous epithelium area is a surrogate for crypt hyperplasia, *p < 0.0001. C) The number of CD3 immuno-positive lymphocytes divided by the total number of villous enterocytes was determined, *p < 0.0001. D) The ratio of the area of villous epithelium divided by the crypt epithelium is a surrogate for the ratio of villus height to crypt depth, *p < 0.0001 [file 13000_2023_1412_MOESM2_ESM.pdf]

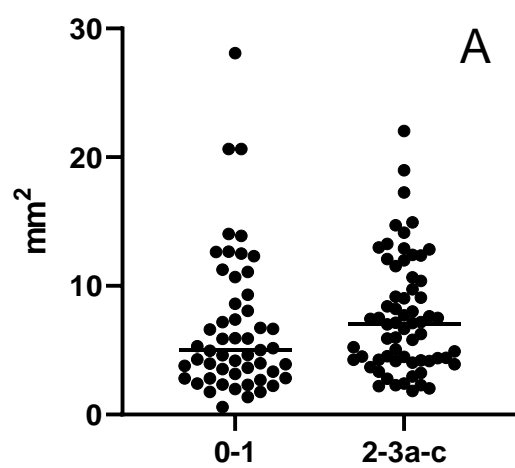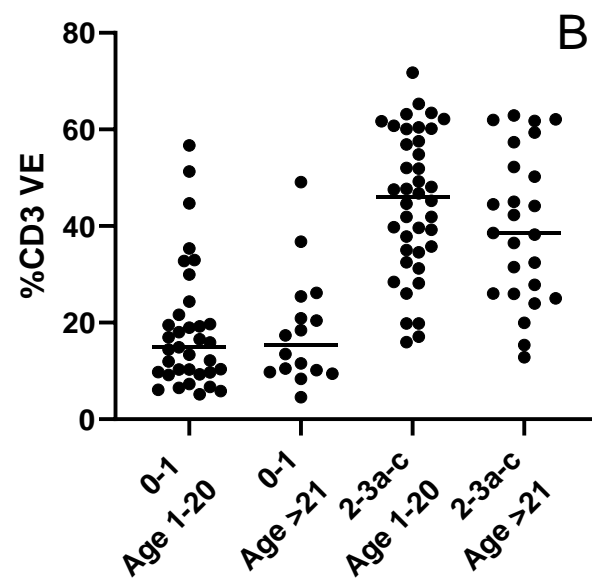

Supplement: Supplementary file 3 — Additional file 3: Supplemental Fig. 3. A) The area of the duodenal tissue biopsy samples was not statistically different across grouped modified Marsh scores. The median area for Type 0–1 scores was 5.0 mm2 and for Type 2-3a-c scores was 7.1 mm2. B) The number of CD3 immuno-positive lymphocytes divided by the total number of villous enterocytes was not statistically different by grouped modified Marsh scores and patient age stratified above and below 21 years. [file 13000_2023_1412_MOESM3_ESM.pdf]

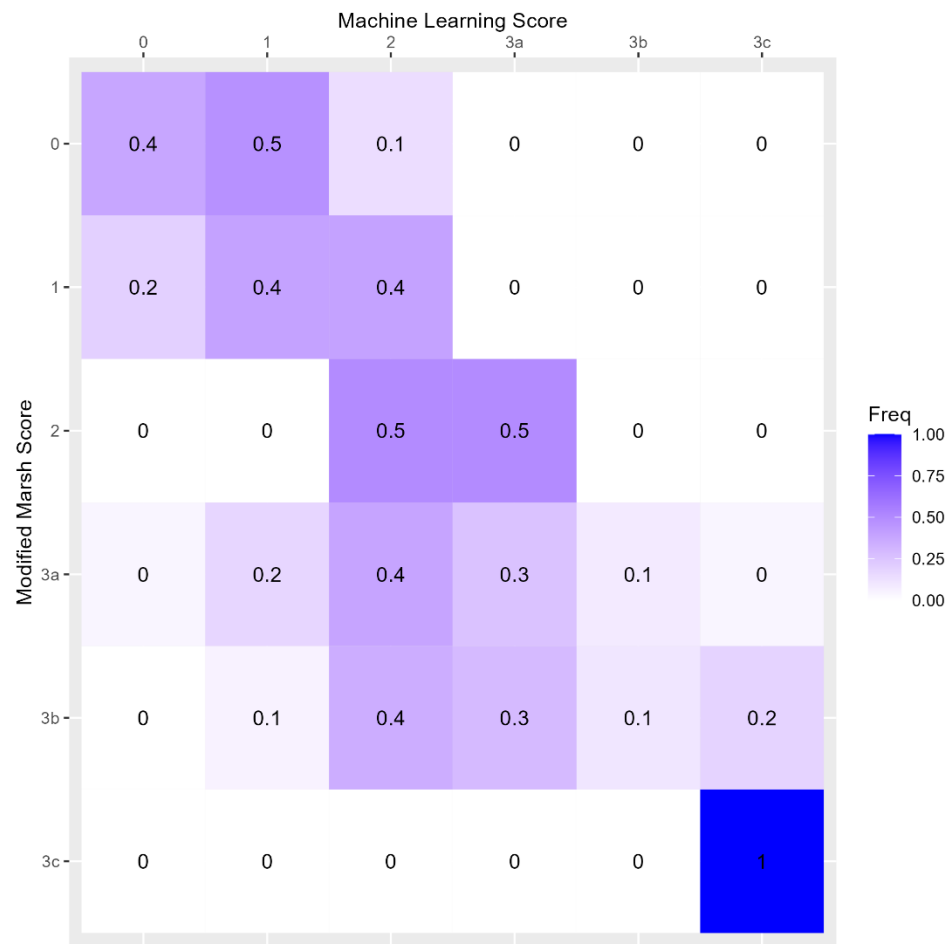

Supplement: Supplementary file 4 — Additional file 4: Supplemental Fig. 4. Confusion matrix heat map showing performance of converted machine learning scores and modified Marsh type. [file 13000_2023_1412_MOESM4_ESM.pdf]
